# P-1099. Post-Pandemic Masking Practices Among Health Care Personnel: Beliefs, Barriers, and Opportunities for Improving Adherence in Clinical Settings

**DOI:** 10.1093/ofid/ofaf695.1294

**Published:** 2026-01-11

**Authors:** Karina Ohri, Samantha E Hanley, Nicholas Allis, Telisa Stewart, Mitchell Brodey, Paul Suits, Stephen J Thomas, Jana Shaw

**Affiliations:** Norton College of Medicine, SUNY Upstate Medical University, Syracuse, NY, USA, fayetteville, NY; Norton College of Medicine, SUNY Upstate Medical University, Syracuse, NY, USA, fayetteville, NY; Norton College of Medicine, SUNY Upstate Medical University, Syracuse, NY, USA, fayetteville, NY; Department of Public Health and Preventive Medicine, SUNY Upstate Medical University, Syracuse, NY, USA, Syracuse, New York; Department of Internal Medicine, SUNY Upstate Community Hospital, Syracuse, NY, USA, Syracuse, New York; Upstate Medical University, Syracuse, New York; Global Health Institute, SUNY Upstate Medical University, Syracuse, New York; Division of Infectious Diseases, Department of Pediatrics, SUNY Upstate Medical University, Syracuse, NY, USA, Syracuse, New York

## Abstract

**Background:**

Hospital-acquired respiratory infections remain a significant portion of healthcare-associated infections. Although facial masking is an evidence-based strategy to reduce transmission, adherence among healthcare personnel (HCP) remains inconsistent. The rationale for post-pandemic masking behavior is not well understood, despite ongoing risk. This study explores gaps in mask-related behaviors, beliefs, and perceptions among HCPs, examines the influence of vaccination status, and identifies opportunities to improve adherence in clinical settings.

**Figure:**
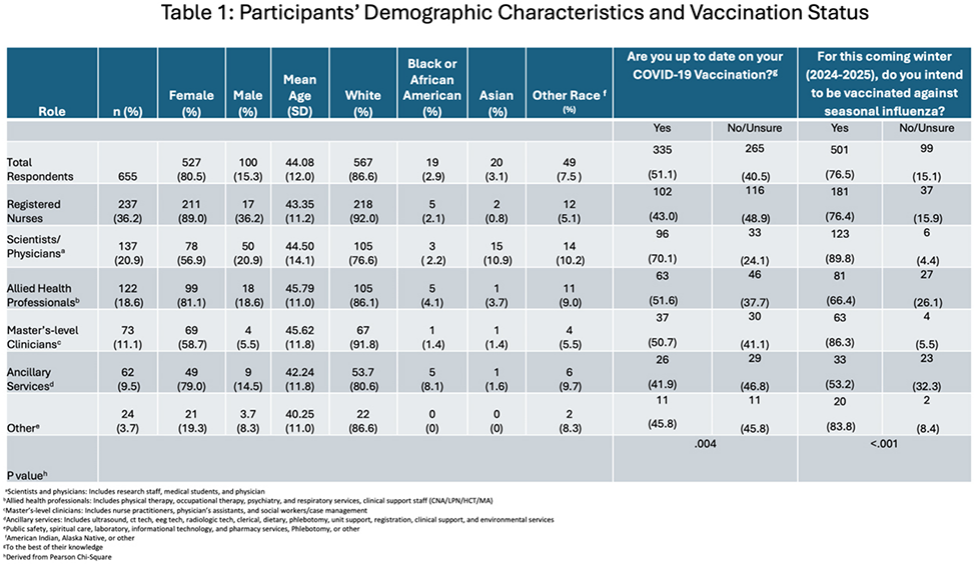

**Figure:**
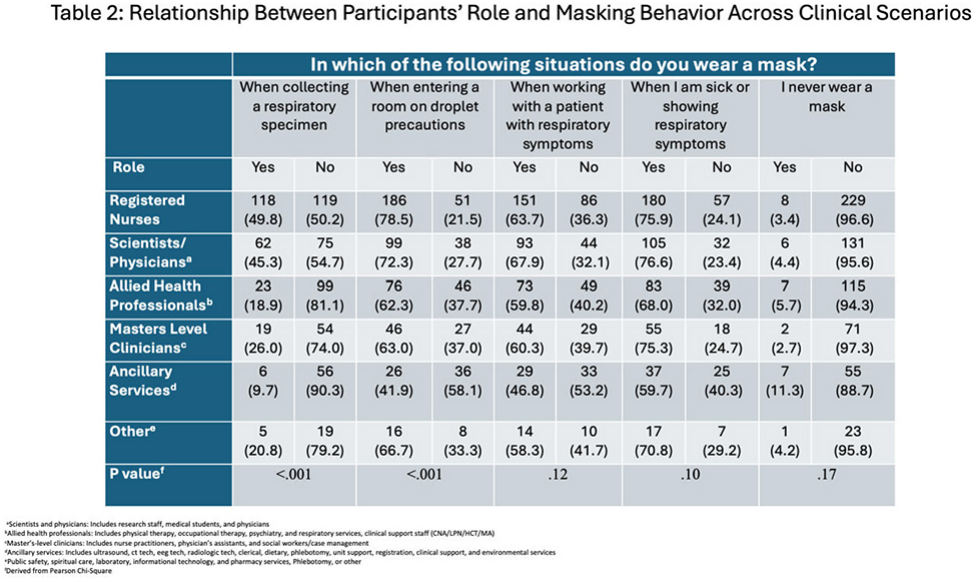
Demographic characteristics of the 655 study participants, stratified by role, along with COVID-19 vaccination status and intention to receive the influenza vaccine.

Mask use was significantly more likely during high-risk activities, particularly when entering droplet precaution rooms.

**Methods:**

A cross-sectional survey was conducted among HCPs providing direct patient care at the State University of New York (SUNY) Upstate Medical University from November 15, 2024, to January 7, 2025. Participation was voluntary and anonymous. The SUNY Upstate IRB deemed the project exempt from review.

**Figure:**
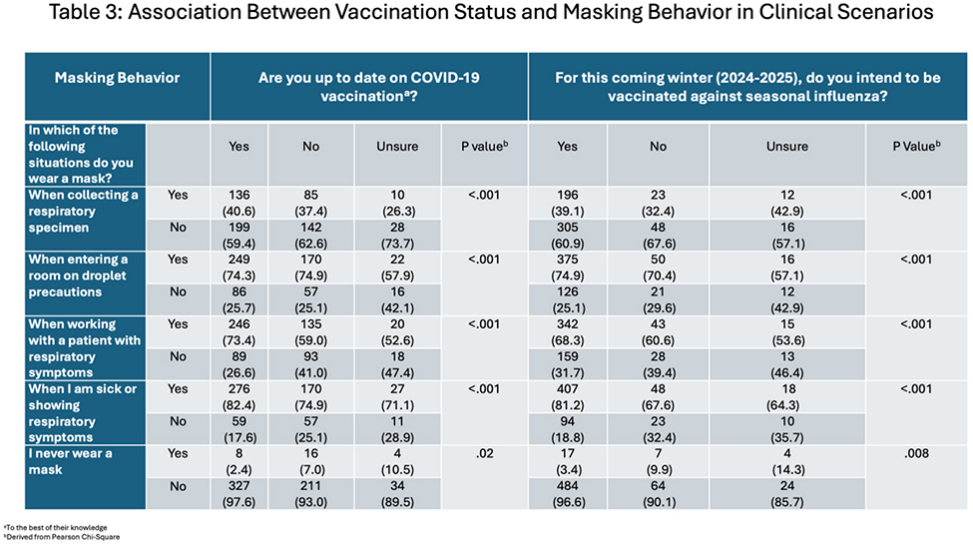

Participants who were up to date on COVID-19 vaccination or intended to receive influenza vaccination were generally more likely to report masking across clinical scenarios, with the exception of situations involving respiratory specimen collection.

**Figure:**
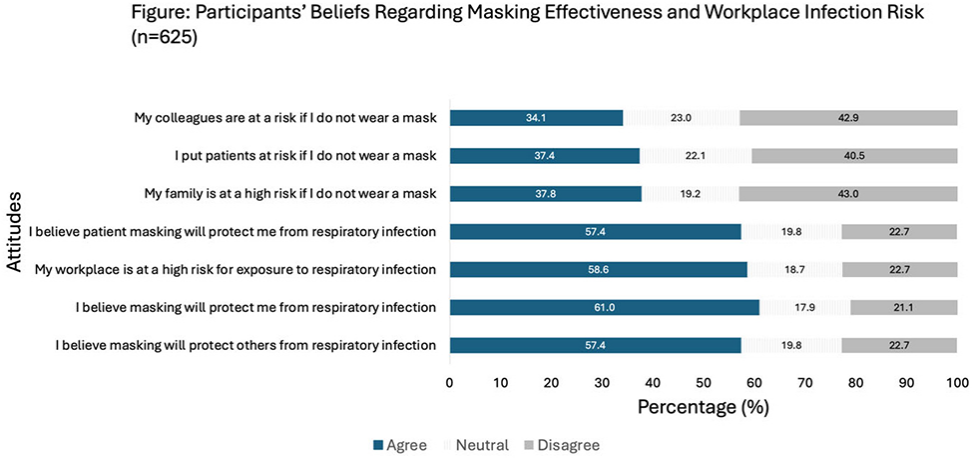

Most participants agreed that masking protects both themselves and others from infection, that the workplace poses a high risk for exposure to respiratory pathogens, and that patient masking reduces the risk of infection.

**Results:**

A total of 655 HCPs responded: registered nurses 237 (36.2%), physicians/scientists 137 (20.9%), allied health professionals 122 (18.6%), master’s-level clinicians 73 (11.1%), and ancillary staff 62 (9.5%). Most participants were white (n=567, 86.6%) and female (n=527, 80.5%), with a mean age of 44.1 years (SD=12). Overall, 355 (51.1%) reported being up-to-date on COVID-19 vaccination, and 501 (76.5%) intended to receive the 2024–2025 influenza vaccine (Table 1). Common barriers to masking included skin irritation (n=206, 31.4%), difficulty breathing (n=196, 30%), and vision interference (n=190, 29%).

Mask use varied by clinical context and role, ranging from 26% to 78.5% with HCPs more likely to wear masks when entering droplet precaution rooms (Table 2). Participants who were up-to-date on COVID-19 vaccination or intended to receive the flu vaccine were generally more likely to mask in clinical settings, except when collecting respiratory specimens (Table 3). Most respondents believed masking protects themselves and others, that the workplace carries high respiratory risk, and that patient masking offers protective benefits (Figure).

**Conclusion:**

Self-reported masking adherence remains suboptimal. Understanding barriers and leveraging leadership, policy, and enforcement are key to promoting consistent, evidence-based masking to protect patients and staff.

**Disclosures:**

Telisa Stewart, DrPH, GSK: Advisor/Consultant Stephen J. Thomas, MD, Icoavax: Advisor/Consultant|Icoavax: Honoraria|Island Pharma: Board Member|Island Pharma: Grant/Research Support|Island Pharma: Stocks/Bonds (Public Company)|Merck: Advisor/Consultant|Merck: Grant/Research Support|Merck: Honoraria|Merck: travel|Moderna: Advisor/Consultant|Moderna: Honoraria|Pfizer: Advisor/Consultant|Pfizer: Honoraria|Pfizer: travel|Primevax: Board Member|Primevax: Stocks/Bonds (Private Company)|Rheonix: Board Member|Rheonix: Stocks/Bonds (Private Company)|Sanofi: Advisor/Consultant|Sanofi: Grant/Research Support|Sanofi: Honoraria|Sanofi: Travel|Takeda: Advisor/Consultant|Takeda: Honoraria|Takeda: travel|Valneva: Advisor/Consultant|Vaxxinity: Advisor/Consultant|Vaxxinity: Honoraria Jana Shaw, MD, MPH, MS, GSK: Advisor/Consultant|Pfizer: Advisor/Consultant

